# An In Vitro–In Vivo Comparison of Two Levodopa Dry Powder Products for Inhalation: A Randomized Trial Comparing Inbrija and Levodopa Cyclops

**DOI:** 10.3390/pharmaceutics17091149

**Published:** 2025-09-02

**Authors:** Julia M. E. Berends, Ettina J. Wimmenhove, Marcel Hoppentocht, Paul Hagedoorn, Henderik W. Frijlink, Floris Grasmeijer

**Affiliations:** 1 Department of Pharmaceutical Technology and Biopharmacy, University of Groningen, Antonius Deusinglaan 1, 9713 AV Groningen, The Netherlands; julia.berends@rug.nl (J.M.E.B.); e.j.wimmenhove@student.rug.nl (E.J.W.); p.hagedoorn@rug.nl (P.H.); h.w.frijlink@rug.nl (H.W.F.); 2 PureIMS, Ceintuurbaan Noord 152, 9301 NZ Roden, The Netherlands; mhoppentocht@pureims.com

**Keywords:** levodopa, Parkinson’s disease, oral inhalation, dry powder for inhalation, dissolution, lung deposition, pharmacokinetics, IVIVC

## Abstract

**Background/Objectives**: The pulmonary administration of levodopa enables a rapid absorption and onset of action, making it a suitable administration route for managing OFF episodes in Parkinson’s disease. Currently, one dry powder product for inhalation (Inbrija) is available on the market, while another (Levodopa Cyclops) is in development. These two products differ substantially in terms of inhaler design, their use and resistance, and their powder formulations. This study aimed to investigate whether these differences translate into in vitro differences in aerosol characteristics and dissolution kinetics and whether any differences were also reflected in the in vivo performance. **Methods**: The in vitro aerosol characteristics were determined via Next Generation Impactor experiments, and the dissolution kinetics were determined with a modified paddle apparatus. A randomized crossover comparative bioavailability study with fasted healthy volunteers was conducted with Inbrija 84 mg and Levodopa Cyclops 45 mg, 90 mg, and 135 mg. **Results**: The results showed similar aerosol characteristics, but Levodopa Cyclops showed substantially faster dissolution behavior than Inbrija. Despite this in vitro difference, the pharmacokinetic profiles of Inbrija 84 mg and Levodopa Cyclops 90 mg were similar, with no differences in C_max_, T_max_, and AUC, showing bioequivalence between the two products. **Conclusions**: This suggests that the systemic absorption of levodopa via the lungs is not limited by dissolution but most likely by its permeation rate. This finding underscores the need to critically apply in vitro tests and critically interpret the results for predicting the in vivo performance of inhaled products.

## 1. Introduction

The pulmonary route of administration has emerged as a promising strategy for the administration of systemically acting drugs, for instance, for the treatment of OFF episodes in Parkinson’s disease. Parkinson’s disease is a progressive neurodegenerative disorder caused by the degeneration of dopaminergic neurons in the substantia nigra. Orally administered levodopa, a precursor of dopamine, is the cornerstone in managing symptoms associated with Parkinson’s disease, such as bradykinesia, tremor, postural instability, rigidity, cognitive impairment, anxiety, and depression [1]. Orally administered levodopa is highly effective at mitigating these symptoms. However, as the disease progresses, this effect wears off, resulting in episodes of poor response (OFF episodes) and episodes of good response (ON episodes) to levodopa. About 4–6 years after starting treatment, approximately 40% of the patients experience these ON/OFF episodes, and after 9–15 years, this is even increased to 70% [2]. OFF episodes may have a sudden onset, can be unpredictable, and can tremendously diminish the quality of life of these patients [3]. Therefore, the rapid termination of OFF episodes is desirable.

Levodopa powder for inhalation was developed as a rescue treatment for OFF episodes in Parkinson’s disease. A major advantage of pulmonary administration over orally administered levodopa is the rapid absorption. The maximum levodopa plasma concentration after inhalation is reached within 15 min [4,5], resulting in a rapid improvement in motor function [6,7]. Approved in 2018 by the Food and Drug Administration [8] and in 2019 by the European Medicines Agency [9], *Inbrija* (Merz Therapeutics) was the first and to date only inhaled levodopa therapy on the market. While Inbrija has expanded the options for treating OFF episodes, offering a choice for a different inhaler type could further enhance the treatment. Providing a variety of inhalers for levodopa would enable a more personalized approach by addressing individual preferences, thereby improving patient satisfaction, medication adherence, and eventually health outcomes [10,11,12]. Moreover, the availability of generic levodopa inhalation products may reduce costs.

In 2015, Luinstra et al. reported on a new levodopa dry powder for inhalation (DPI) product, currently known as *Levodopa Cyclops* (PureIMS) [13]. Levodopa Cyclops and Inbrija differ significantly regarding their excipients, powder production methods, the resulting powder morphology, and powder density, as well as the design of the inhaler devices, their resistances, and their instructions for use. Table 1 provides an overview of the differences between Inbrija and Levodopa Cyclops. Separate studies demonstrated that both levodopa used with Inbrija and used with Cyclops are rapidly absorbed [4,5,14] and are effective in the termination of OFF episodes [6,7,15]. However, to date, no pharmacokinetic, efficacy, and usability studies were conducted comparing the two products head-to-head.

Inhaled drug products are relatively complex due to the interplay between the formulation, the device, and the patient’s breathing profile. This complexity is particularly relevant for bioequivalence testing, as it implies that the pharmacokinetics are not solely determined by the formulation itself. In this randomized crossover study, we compare the pharmacokinetics of Inbrija and Levodopa Cyclops in healthy volunteers. Additionally, we assessed the in vitro dissolution and aerosol characteristics of Inbrija and Levodopa Cyclops since, based on the differences between the products, we expect to find in vitro differences that may reflect in the in vivo pharmacokinetic data.

## 2. Materials and Methods

### 2.1. Materials

A 0.1 M HCl solution (pH 1.0) was prepared by diluting 37% HCl (Merck, Darmstadt, Germany) with demineralized water. Phosphate-buffered saline (pH 7.4) was composed of 137 mM NaCl (Boom, Meppel, The Netherlands), 2.7 mM KCl (Boom, The Netherlands), 10 mM Na_2_HPO_4_ (as dihydrate; Merck, Germany), and 1.8 mM KH_2_PO_4_ (Boom, The Netherlands) in demineralized water. Reduced glutathione and ethylenediaminetetraacetic acid (EDTA) were obtained from Merck (Germany). Whatman glass microfiber grade GF/A filters (⌀ 50 mm; Merck, Germany) had a nominal pore size of 1.6 μm. Inbrija was obtained from Myonex (Berlin, Germany), and Levodopa Cyclops was obtained from PureIMS (Roden, The Netherlands). Calibration curves were prepared using the Ph. Eur. Reference Standard of levodopa. The Levodopa Cyclops and Inbrija batches used for the in vitro characterization were the same as those used for the in vivo pharmacokinetic study.

### 2.2. In Vitro Characterization

#### 2.2.1. Content Analysis

The content of five individual capsules for Inbrija and the content of five individual dose compartments for Levodopa Cyclops were weighed and dissolved in 0.1 M HCl in a measuring flask. The absorbance was measured spectrophotometrically at 280.6 nm (Genesys 150; Thermo Scientific, Zaventem, Belgium), after confirming that the excipients within the formulations did not affect the analysis. The levodopa content was calculated using a calibration curve.

#### 2.2.2. Aerosol Characterization

A Next Generation Impactor (NGI; Copley Scientific, Nottingham, UK) with a standard stainless steel induction port without a pre-separator was used to characterize the aerodynamic particle size distribution of Inbrija and Levodopa Cyclops. To minimize bounce effects, stages 1 to 7 of the NGI were covered with Whatman glass microfiber, each coated with 1 mL of 0.1 M HCl. The measurements were conducted at the same pressure drops for Inbrija and Levodopa Cyclops, despite the difference in inhaler resistance between both devices. This was based on a study by Luinstra et al. (2015), who concluded that the maximum pressure drop patients achieve is independent of the inhaler resistance [24]. Pressure drops of 2, 4, and 6 kPa were chosen, corresponding to flow rates of 23, 33, and 42 L_s_/min for Inbrija (inhaler resistance = 0.0671 kPa^0.5^⋅min⋅L_n_^−1^) and 40, 57, and 72 L_s_/min for Levodopa Cyclops (inhaler resistance = 0.0390 kPa^0.5^⋅min⋅L_n_^−1^). Inhalation times were chosen to correspond to an inhaled volume of 4 L, as described in the 11th edition of the Ph. Eur. The labeled levodopa dose was 42 mg per capsule for Inbrija and 45 mg per device for Levodopa Cyclops. For Inbrija, five replicates were measured at each pressure drop; for Levodopa Cyclops, the measurement was performed in duplicate at each pressure drop due to the limited number of Levodopa Cyclops available from the clinical batch. The powder retained in the inhaler devices and the powder deposited onto the NGI stages were recovered in a known volume of 0.1 M HCl. The solutions were analyzed spectrophotometrically (Genesys 150; Thermo Scientific, Belgium) at 280.6 nm. The emitted dose and NGI stage depositions were calculated using a calibration curve and corrected for the recovered fraction. The fine particle dose (<5 μm) was determined by interpolating the cumulative aerodynamic particle size distribution curves.

#### 2.2.3. Dissolution

The in vitro dissolution of the Levodopa Cyclops and Inbrija formulations was assessed using a modified USP Apparatus 2 (Sotax AT 7, Aesch, Switzerland). An in-house developed 3D-printed DPI filter holder for dissolution testing was used to add the DPI formulations to the dissolution vessels. This filter holder was printed with gray resin V4 using an SLA 3D printer (Form 3+, Formlabs, Somerville, MA, USA). It was designed to prevent an insufficiently stirred dead zone underneath the filter holder. A quantity of the formulations corresponding to 10 ± 0.2 mg of levodopa was applied onto a Whatman glass microfiber filter: 11.5 mg for Inbrija and 10.1 mg for Levodopa Cyclops, based on levodopa contents of 87.1% (SD = 0.1%) and 98.7% (SD = 0.3%) for Inbrija and Levodopa Cyclops, respectively. The glass microfiber filter was inserted into the bottom plate of the filter holder. Subsequently, the powder was sandwiched by another glass microfiber filter, after which the other part of the filter holder was placed on top. The filter holder was firmly closed using stainless steel wing nuts and bolts and placed in a dissolution vessel containing 600 mL of phosphate-buffered saline of pH 7.4 ± 0.1 with 100 μM reduced glutathione and 30 μM EDTA. Levodopa is prone to oxidation, especially at this pH [25]. Without the addition of the antioxidants, the dissolution medium turned black, and the levodopa concentration dropped during the measurements, which indicates oxidation of levodopa. Sink conditions were maintained throughout the experiment. All dissolution measurements were performed at 37 ± 0.5 °C with a stirring speed of 140 rpm. Twelve replicates were measured for each formulation. The absorbance at 280.6 nm was measured for 2 h with an in-line UV-Vis spectrophotometer (Evolution 300 UV-Vis spectrophotometer, Thermo Fisher Scientific, Waltham, MA, USA) with a sampling interval of 2 min. The filter holder, including wing nuts and bolts, did not affect the analysis. To evaluate the similarity between the dissolution profiles, the difference factor (f1) and similarity factor (f2) were calculated with Inbrija as the reference product. Similarity was established when the f1-value was below 15 and the f2-value was above 50.

### 2.3. In Vivo Comparative Bioavailability Study

#### 2.3.1. Design, Participants, and Selection Criteria

This phase 1, open-label, randomized, crossover, comparative bioavailability study was conducted at the Diagnostic & Consultative Centre Ascendent Ltd. in Sofia, Bulgaria, between 18 September and 9 November 2023. The study was registered in ClinicalTrials.gov (NCT06037590) and complied with the Clinical Trial Regulation (CTR) and the Declaration of Helsinki and its subsequent amendments (Fortaleza, Brazil, October 2013). The Bulgarian Drug Agency approved the study (ИАЛ-22521) following submission to the Clinical Trials Information System (CTIS). All participants provided written informed consent before participating in the study. The first subject signed informed consent on 18 September 2023, and the last study visit was on 9 November 2023.

The sample size for this clinical trial was set at 26 participants. This sample size was not based on a formal power calculation. The most relevant inclusion and exclusion criteria are presented here. Eligible participants were males and non-pregnant, non-lactating females aged between 18 and 55 years, who were non-smokers for at least six months, physically and mentally healthy, had a body mass index within the range of 18.0 to 30.0 kg/m^2^, and exhibited normal spirometry values (forced expiratory volume in 1 s (FEV_1_) and forced vital capacity (FVC)). Exclusion criteria included a history or presence of cardiovascular, renal, hepatic, pulmonary, metabolic, endocrine, hematological, gastrointestinal, neurological, or psychiatric diseases, as well as abnormalities in vital signs or laboratory values. Intake or administration of enzyme-inducing, organ-toxic, or long half-life drugs was prohibited within four weeks prior to the screening visit.

#### 2.3.2. Study Procedures and Medication

Thirty participants were screened, of whom 26 were enrolled in the study. The participants received 45 mg, 90 mg, and 135 mg levodopa in one to three 45 mg inhalations via pre-filled Levodopa Cyclops devices, and 84 mg levodopa in two 42 mg inhalations via an Inbrija device. The study was performed under fasting conditions on four consecutive days, according to a randomized crossover scheme. The subjects were randomly assigned to one of the four administration sequences using the Oracle Clinical 5.2.2 randomization module. On day 0 and prior to dosing on days 1 to 4, participants received training on properly using the Inbrija and Levodopa Cyclops devices. One hour before each treatment, participants were given an oral dose of 50 mg carbidopa to prevent levodopa-induced nausea and vomiting, and to more accurately simulate real-world use of inhaled levodopa as an add-on therapy to standard oral levodopa/decarboxylase inhibitor therapy. Blood samples were collected via an intravenous catheter into EDTA tubes at 60, 30, and 15 min prior to treatment and at 3, 6, 9, 12, 15, 18, 21, 24, 27, 30, 40, and 60 min, as well as 1.5, 2, 4, and 8 h post-dosing. The levodopa plasma concentrations were determined by QPS Netherlands using a validated LC-MS/MS method.

#### 2.3.3. Objectives and Pharmacokinetic Analysis

The primary objective of this clinical trial was to identify the dose at which the comparative bioavailability of levodopa between Inbrija and Levodopa Cyclops was achieved. This was assessed based on the maximum plasma concentration (C_max_), the area under the plasma concentration/time curve from time 0 to 4 h (AUC_0–4_), the area under the plasma concentration/time curve from time 0 h to the last measured concentration (AUC_0–t_), and the area under the plasma concentration/time curve from time 0 h extrapolated to infinity (AUC_0–∞_). The area under the curve was calculated using the linear trapezoid method, with baseline levels normalized to zero by taking the mean of the three pre-dose levodopa plasma samples. Moreover, the time to C_max_ (T_max_) and the elimination half-life (t_1/2_) were evaluated. The secondary objective was to compare the safety profiles of Inbrija and Levodopa Cyclops. Safety was evaluated by questioning the participants about adverse events and by measuring vital signs (blood pressure and pulse rate) 50 min post-dose. Additionally, clinical laboratory values were measured at screening and after the final treatment visit to further evaluate the safety of inhaled levodopa.

#### 2.3.4. Statistics

The normality of the pharmacokinetic parameters was checked with a Shapiro–Wilk test. When the data followed a normal distribution (*p* > 0.05), the mean with standard deviation (SD) was reported to describe the data. When the data did not follow a normal distribution, the median (Q1–Q3) was used to describe the data. To evaluate bioequivalence between Inbrija and the different doses of Levodopa Cyclops, the C_max_, AUC_0–4_, AUC_0–t_, and AUC_0–∞_ were compared and assessed based on the EMA Guidelines [26]. First, a General Linear Model Analysis of Variance (GLM-ANOVA) was performed based on the logarithmically transformed geometric means. The GLM-ANOVA was fitted with the treatment, the period (4 levels), the sequence of the treatment (4 levels), and the participant nested in sequence. Based on the mean squared error from the GLM-ANOVA, 90% confidence intervals were constructed for the ratio of Inbrija to each Levodopa Cyclops dose. Finally, the confidence intervals were back-transformed to non-logarithmic values. Bioequivalence was demonstrated when the confidence intervals lied within the limits of 80.00 to 125.00%. Additionally, to compare the variance in levodopa plasma concentration between Inbrija and Levodopa Cyclops 90 mg, a Levene’s test was performed on the log-transformed plasma concentrations for each time point. A *p*-value below 0.05 was considered statistically significant.

## 3. Results

### 3.1. In Vitro Characterization

#### 3.1.1. Aerosol Characterization

**Figure 1:**
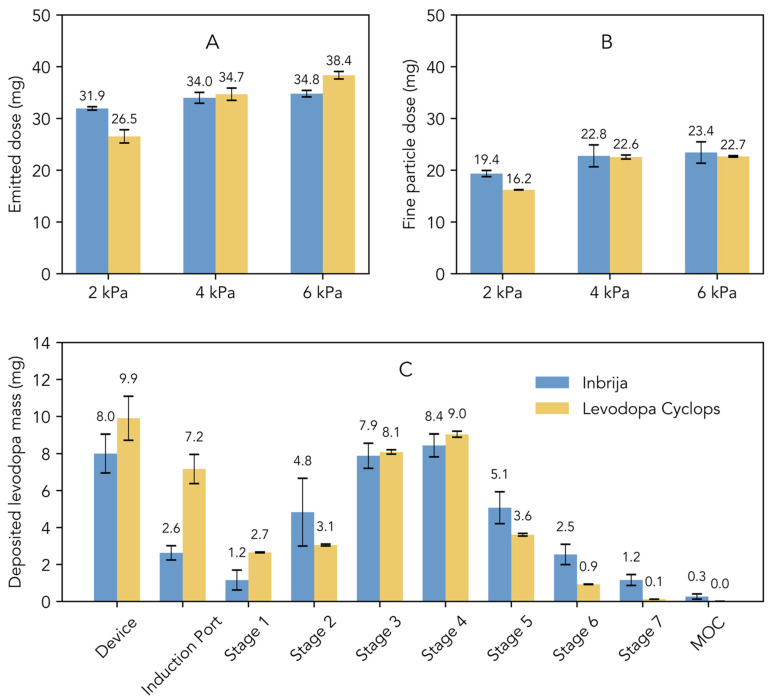
Comparison of the in vitro aerosol characteristics of Inbrija (labeled dose of 42 mg) and Levodopa Cyclops (labeled dose of 45 mg) obtained via Next Generation Impactor measurements. Data are expressed as means with standard deviations for Inbrija (*n* = 5) and means with min and max values for Levodopa Cyclops (*n* = 2). (**A**) The emitted dose, defined as the total amount of levodopa released from the device. (**B**) The fine particle dose, defined as the dose of particles with an aerodynamic diameter of 5 μm or smaller. (**C**) Stage-by-stage comparison at a pressure drop of 4 kPa. MOC = micro-orifice collector.

Figure 1A illustrates the emitted dose, which represents the total amount of levodopa released from the device. At 2 kPa, the emitted dose from Inbrija was higher compared to that from Levodopa Cyclops. However, Levodopa Cyclops exhibited a greater dependency on pressure drop, leading to a comparable emitted dose at 4 kPa and a higher emitted dose at 6 kPa compared to Inbrija. Figure 1B illustrates the fine particle dose, which refers to the dose of particles with an aerodynamic diameter of 5 μm or smaller. At 2 kPa, Inbrija exhibited a higher fine particle dose than Levodopa Cyclops. The fine particle dose of Levodopa Cyclops increased significantly from 2 kPa to 4 kPa. The same effect was observed for Inbrija, although less pronounced. At 4 kPa, the fine particle doses of Inbrija and Levodopa Cyclops were comparable. Notably, at 6 kPa, the fine particle doses of Inbrija and Levodopa Cyclops were similar to those observed at 4 kPa.

**Figure A1:**
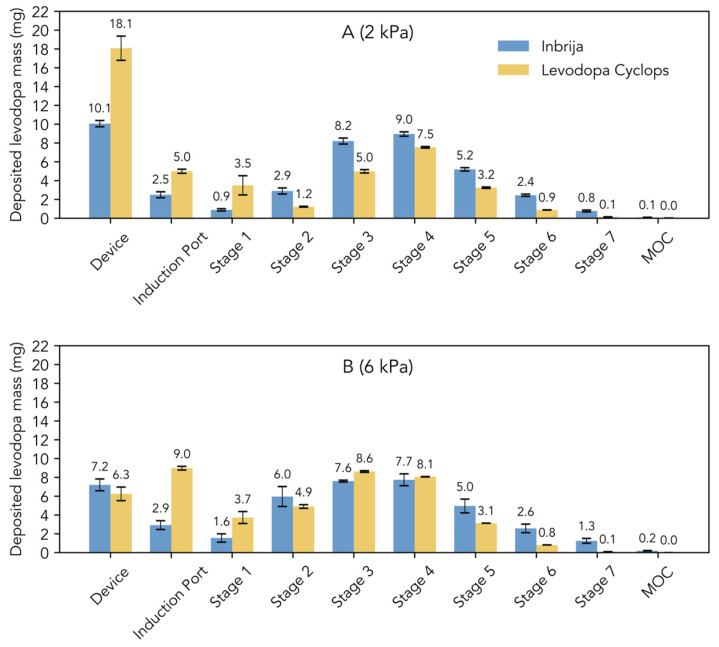
Stage-by-stage comparison of the Next Generation Impactor experiments with Inbrija (42 mg) and Levodopa Cyclops (45 mg) at (**A**) 2 kPa and (**B**) 6 kPa. Data are expressed as means. Error bars represent standard deviations for Inbrija (*n* = 5) and min and max values for Levodopa Cyclops (*n* = 2). MOC = micro-orifice collector.

Figure 1C shows the stage-by-stage comparison of Inbrija and Levodopa Cyclops at 4 kPa. Compared to Inbrija, Levodopa Cyclops exhibited higher device retention and deposition in the induction port. In contrast, Inbrija demonstrated higher depositions from stage 5 (cut-off diameter of 1.28 µm for Inbrija and 0.96 µm for Levodopa Cyclops) to the micro-orifice collector, with 9.1 mg (SD = 1.7 mg) for Inbrija and 4.7 mg (SD = 0.1 mg) for Levodopa Cyclops. Similar trends were observed in the stage-by-stage comparisons at 2 and 6 kPa (Appendix A Figure A1), with the exceptions that Levodopa Cyclops showed a more pressure drop-dependent device retention (inversely related) and deposition in the induction port (positively related) than Inbrija.

#### 3.1.2. Dissolution

**Figure 2:**
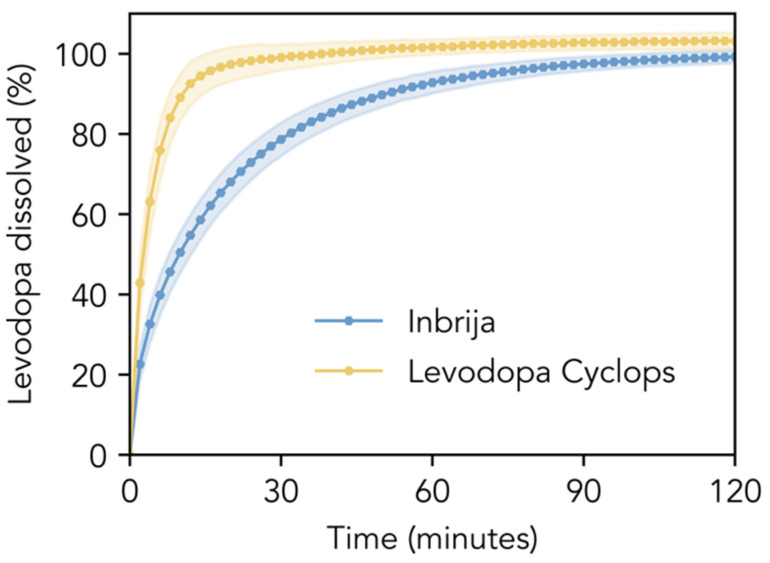
In vitro dissolution profiles. Dissolution profiles of the Inbrija and Levodopa Cyclops powder formulations (amounts corresponding to a levodopa mass of 10 mg) in 600 mL of phosphate-buffered saline pH 7.4 with 100 μM reduced glutathione and 30 μM EDTA. Data are expressed as means with the shaded areas representing standard deviations (*n* = 12).

Figure 2 compares the dissolution profiles of the Inbrija and Levodopa Cyclops powder formulations in phosphate-buffered saline pH 7.4. The dissolution of Inbrija was relatively slow; 80% of the levodopa within the formulation dissolved within 33.0 min (SD = 5.4 min). In contrast, Levodopa Cyclops exhibited a significantly faster dissolution rate: 80% of the levodopa dissolved within 8.3 min (SD = 1.7 min). The f1- and f2-values were 29.5 and 30.7, respectively, indicating that the dissolution profiles are significantly different.

### 3.2. In Vivo Comparative Bioavailability Study

#### 3.2.1. Demographics

**Figure 3:**
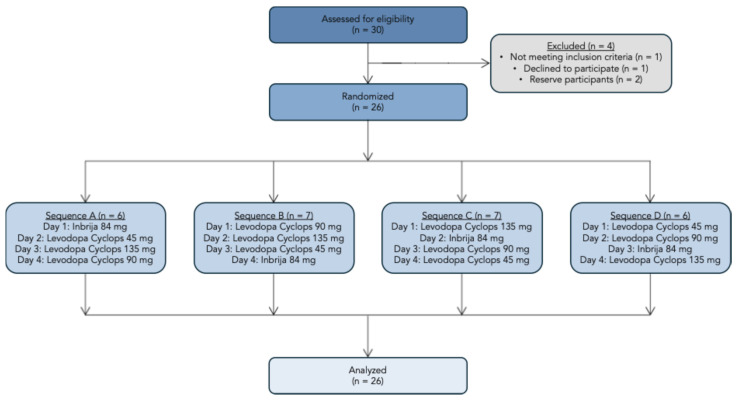
Study flowchart.

We screened 30 participants, of whom 26 were enrolled in the study. One participant failed the screening, one decided to discontinue the participation, and two were reserve participants. The study flowchart is presented in Figure 3. Amongst the 26 participants were 9 (34.6%) females and 17 (65.4%) males. The mean age was 36.9 years (SD = 11.3 years), and the mean body mass index was 23.6 kg/m^2^ (SD = 3.19 kg/m^2^).

#### 3.2.2. Pharmacokinetics

**Figure 4:**
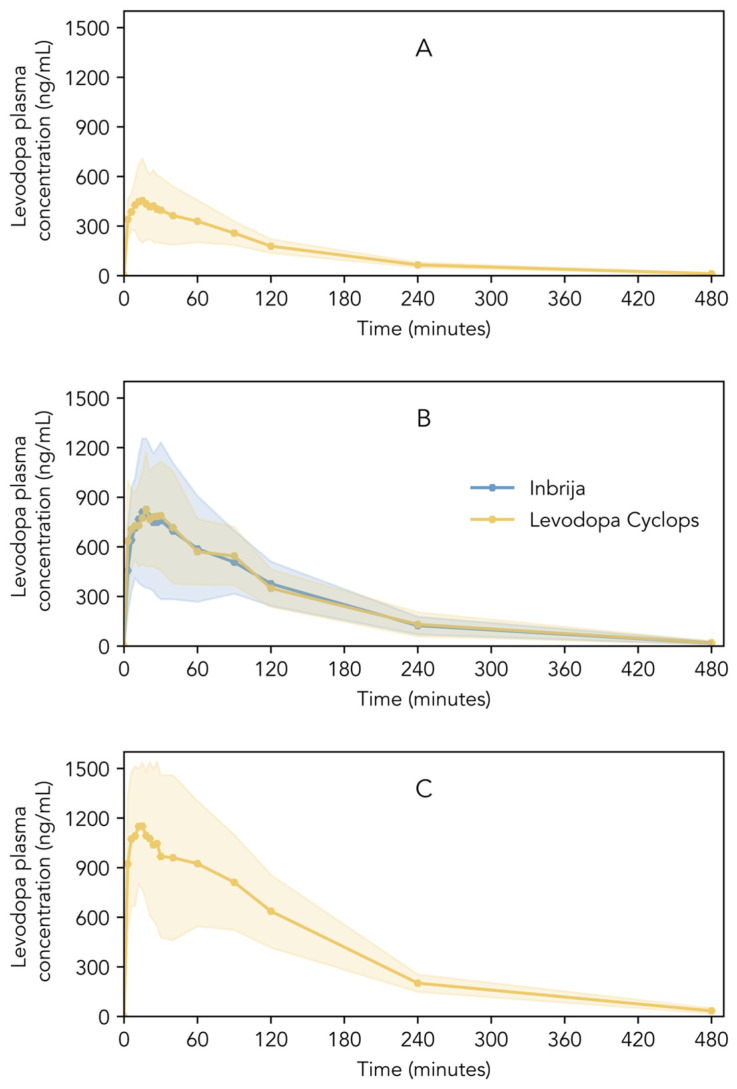
Levodopa plasma concentrations of healthy volunteers after the inhalation of (**A**) Levodopa Cyclops 45 mg, (**B**) Inbrija 84 mg and Levodopa Cyclops 90 mg, and (**C**) Levodopa Cyclops 135 mg. Data are expressed as means with the shaded areas representing standard deviations (*n* = 26).

Figure 4A–C show the pharmacokinetic profiles of Inbrija and Levodopa Cyclops. Descriptive statistics of C_max_, T_max_, AUC_0–4_, AUC_0–t_, AUC_0–∞_, and t_1/2_ are shown in Table 2. Inbrija and all three Levodopa Cyclops doses showed a rapid increase in levodopa plasma concentration immediately after inhalation; C_max_ was reached between 15 and 24 min (median), depending on the treatment. Inbrija and Levodopa Cyclops 90 mg showed similar pharmacokinetic profiles (Figure 4B). Moreover, the 90% confidence intervals of the ratio Levodopa Cyclops 90 mg to Inbrija of C_max_ (93.36–119.32%), AUC_0–4_ (92.68–120.06%), and AUC_0–∞_ (91.98–123.72%) lied within the 80.00–125.00% range, establishing bioequivalence between Inbrija and Levodopa Cyclops 90 mg. Only the confidence interval of the AUC_0–t_ (91.34–125.28%) was slightly beyond the bioequivalence criterion. The Levene’s test indicated that the variance in levodopa plasma concentrations between Inbrija and Levodopa Cyclops 90 mg was statistically equivalent at most time points (*p* > 0.05). However, statistically significant differences in variance were observed at three time points: 21 (*p* = 0.02), 24 (*p* = 0.04), and 30 min (*p* = 0.04), where Levodopa Cyclops showed lower variance compared to Inbrija.

#### 3.2.3. Safety and Tolerability

No serious adverse events were observed during the study period in any of the 26 subjects. Six non-serious adverse events were registered. One participant suffered from mild diarrhea after the Levodopa Cyclops 45 mg treatment, and another participant experienced a mild cough after the Inbrija treatment, which were both judged to be related to the treatment. One participant had mild deviations of the hematological values after the Inbrija treatment, which was judged not to be related to the treatment.

## 4. Discussion

In this study, we investigated the in vitro dissolution and aerosol characteristics of Inbrija and Levodopa Cyclops to assess whether differences in product characteristics resulted in in vitro differences. Furthermore, we compared the pharmacokinetics of Inbrija and Levodopa Cyclops to evaluate whether possible in vitro differences resulted in differences in in vivo pharmacokinetics. To our knowledge, this is the first study investigating both the in vitro and the in vivo characteristics of DPIs intended for systemic drug administration, in one single study, using the same batch for the in vitro and the in vivo studies, thereby excluding batch-to-batch variation.

The aerosol characteristics are an important factor influencing the systemic drug absorption rate of dry powders for inhalation, as they determine the site of deposition within the respiratory tract [27]. Particles with a small aerodynamic diameter tend to deposit in the alveolar region, where the extensive surface area, thin epithelial barrier, and extensive vascularization provide an ideal environment for rapid absorption of drugs [28]. In contrast, particles with a larger aerodynamic diameter tend to deposit higher in the respiratory tract, where absorption is slower. For instance, for fluticasone propionate, a biphasic absorption pattern was demonstrated, with 38- to 62-fold slower absorption from the central lungs compared to the peripheral lungs [29]. The physicochemical properties of the powder formulation (e.g., primary particle size distribution and the adhesive and cohesive behavior of the particles), as well as the device’s ability to deagglomerate the powder formulation, are determinants of the aerosol characteristics and thereby determine the site of deposition within the respiratory tract. Additionally, the device’s resistance (determining inhalation flow rate and duration) and a patient’s breathing abilities also influence the deposition within the respiratory tract. Inbrija and Cyclops, being high-resistance and medium-to-high-resistance devices, respectively, both generate relatively gradual and low airflow rates, which favor deep lung deposition [30]. Despite the devices and the powder formulations being substantially different, the NGI results showed no substantial differences between Inbrija and Levodopa Cyclops regarding the emitted dose and fine particle dose. This can be explained by the fact that the extent of aerosolization is determined by the geometric particle size distribution, density, and morphology of the powders (Table 1), in combination with the inhaler’s dispersion efficiency. Thus, aerosolization may still be similar, even though the products differ in these four aspects. However, the stage-by-stage deposition showed slightly more pronounced differences between Inbrija and Levodopa Cyclops. Levodopa Cyclops showed higher deposition in the induction port than Inbrija, suggesting greater oropharyngeal deposition for Levodopa Cyclops. Furthermore, when combining stages 5, 6, and 7—the stages that represent deep lung deposition—Inbrija demonstrated a slightly higher dose of fine particles than Levodopa Cyclops, indicative of greater peripheral lung delivery for Inbrija.

Another factor that may influence the absorption rate of dry powders for inhalation is the dissolution rate. Based on the formulations, we hypothesized that Levodopa Cyclops would show faster dissolution compared to Inbrija. The main excipient in the Inbrija formulation is DPPC. DPPC is a phospholipid with a hydrophilic head group and two long hydrophobic fatty acid tails, making it extremely poorly water-soluble. The poor solubility of DPPC likely decreases the dissolution rate of the Inbrija formulation. L-leucine, in contrast, is an amino acid with a hydrophobic side chain, but the amino and carboxyl groups are hydrophilic. Although L-leucine is not extremely water-soluble, its aqueous solubility is significantly higher than that of DPPC. Moreover, L-leucine only accounts for 2% of the Levodopa Cyclops formulation, whereas DPPC accounts for 8% of the Inbrija formulation. Indeed, we observed a noticeable difference in in vitro dissolution profiles between Inbrija and Levodopa Cyclops: Levodopa Cyclops dissolved markedly faster than Inbrija.

Based on the in vitro aerosol characteristics, no major differences in the in vivo pharmacokinetic profiles would be expected, although a slightly faster absorption could be anticipated for Inbrija, because of an expected slightly higher deep lung deposition. Based on the in vitro dissolution rates, we expected faster absorption of Levodopa Cyclops than of Inbrija. Interestingly, the in vitro differences did not translate into in vivo differences. The in vivo data demonstrated comparative bioequivalence between Inbrija and Levodopa Cyclops 90 mg and showed similar pharmacokinetic profiles, without statistically significant differences in C_max_, T_max_, and AUC. Moreover, the equivalent or, at certain time points, lower variances for Levodopa Cyclops indicate that its levodopa plasma concentrations are at least as consistent as those of Inbrija. The Levodopa Cyclops dose optimized for bioequivalence to Inbrija would be 84 mg, and hence, equal to the Inbrija dose. One explanation might be that the aerosol characteristics, favoring faster absorption for Inbrija, and the dissolution rate, favoring faster absorption for Levodopa Cyclops, offset each other. Another possible explanation may lie in the properties of the API, as also suggested by the principles of the Inhalation-Based Biopharmaceutics Classification System (iBCS) [31,32,33,34]. The dissolution rate of rapidly dissolving APIs, including those classified as iBCS Class I and III, may be relatively insensitive to formulation characteristics, as their rate of absorption may not necessarily be limited by the dissolution rate. It could be that the dissolution of the Inbrija formulation, although slower than Levodopa Cyclops, is sufficiently fast to saturate the permeation across the pulmonary epithelium. This would imply that the absorption rate of levodopa is permeability-limited, rather than solubility-limited. Considering the chemical properties of levodopa, this appears plausible. Levodopa is slightly soluble in water, but its zwitterionic nature at physiological pH and high polarity [35] may slightly reduce the passive transport rate across the epithelium.

While the findings provide valuable insights, certain methodological limitations should be acknowledged. Although NGI is a widely used and accepted method to assess the aerodynamic properties of orally inhaled drug products (OIDPs), it has some limitations when applied to estimate the deposition in the lungs. Firstly, a straightforward comparison of the performance of OIDPs is complicated when the inhaler resistances and dispersion mechanisms are different. The pressure drops applied during NGI testing may not reflect the pressure drops with which a specific device is typically used. Secondly, NGI does not consider powder emission time, which is a determinant of regional lung deposition. On the other hand, we conducted some preliminary measurements on powder emission via time-sliced laser diffraction and could not find a substantial difference between both products. Thirdly, the flow rate within an NGI is constant, unreflective of a real inhalation maneuver. Additionally, it does not take variability in breathing profiles amongst humans into account, unless a breathing simulator is used. Gobetti et al. demonstrated that in vitro–in vivo correlations based on a realistic breathing profile were predictive of pharmacokinetic outcomes, whereas conventional NGI testing failed to show such predictive value [36]. Fourthly, the NGI is designed to be a robust tool and does, therefore, not resemble the complex anatomy and physiology of the respiratory tract. It does not consider the hygroscopicity of particles, interactions of particles with the epithelial lining fluid, branching within the respiratory tract, and airflow recirculation. Lastly, during NGI testing, bounce effects may occur, especially during characterization of high-dose aerosols [37]. This can lead to overestimation of the fine particle dose. Despite our efforts, we could not fully eliminate bounce effects in this study, which were most notably present with Inbrija and evidenced by visual powder deposition to the underside of the NGI nozzles. This may have caused a slight bias towards finer particle sizes, particularly for Inbrija. A limitation of the in vitro dissolution method is that it may have insufficiently reflected the low volume, surfactant-rich environment of the epithelial lining fluid, of which the composition and volume are, furthermore, dependent on the region within the pulmonary tract [38]. Finally, Inbrija showed slower dissolution kinetics in vitro, but in vivo, the high concentration of surfactants in the epithelial lining fluid may be sufficient to dissolve the formulation rapidly, resulting in a comparable dissolution rate to Levodopa Cyclops.

The findings of this study suggest that differences in dry powder for inhalation products can lead to differences in in vitro characteristics; however, these in vitro characteristics do not necessarily translate into differences in in vivo pharmacokinetics. We established bioequivalence across Inbrija and Levodopa Cyclops, underscoring that the pharmacokinetic profiles can be similar even when the inhalation product characteristics and in vitro performance diverge. This implies that the use of in vitro methods to predict the pharmacokinetics of inhalation products may give rise to incorrect conclusions when the predictive power of the in vitro method has not been sufficiently validated. Further research is essential to establish standardized in vitro methods suitable for the development, regulatory approval, or quality control of (generic) inhalation products, in which the objective of the in vitro test method should guide test design.

## Figures and Tables

**Table 1:** Product differences between Inbrija and Levodopa Cyclops.

	Inbrija	Levodopa Cyclops
Levodopa content	90% *w*/*w* [16]	98% *w*/*w* [13]
Excipients	8% *w/w* 1,2-dipalmitoyl-sn-glycero-3-phosphocholine (DPPC) and 2% *w*/*w* NaCl [16,17]	2% *w*/*w* L-leucine [13]
Additional excipients	N/A	Sweeper lactose (lactose 250–315 μm) ^1^ [18]
Powder production method	Spray-drying [19]	Spiral jet-milling [13]
Particle morphology ^2^	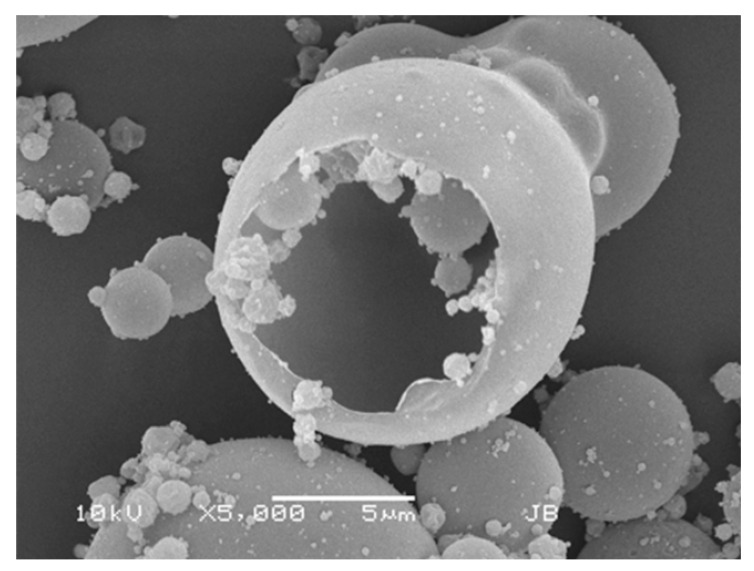 Spherical, hollow particles	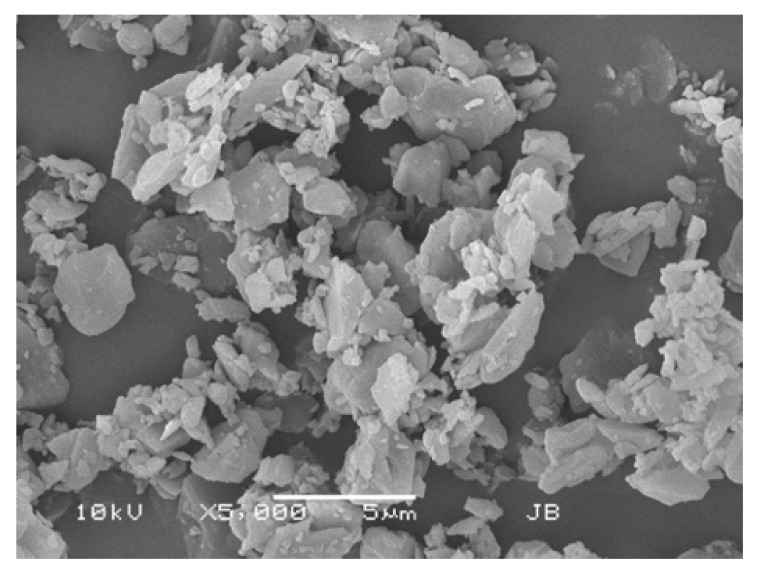 Irregularly shaped, solid particles
Inhaler resistance ^2^	0.0671 kPa^0.5^⋅min⋅L^−1^	0.0390 kPa^0.5^⋅min⋅L^−1^
Single-/multiple-use device	Multiple [20]	Single [21]
Powder loading	Capsules [20]	Pre-filled dose compartment [21]
Dispersion principle	Spinning of the capsule [22]	Air classifier technology [23]
Labeled dose	84 mg per dose (1 dose = 2 capsules) [20]	To be determined
Delivered dose	66 mg per dose (1 dose = 2 capsules) [20]	To be determined

^1^ Sweeper lactose consists of coarse lactose particles, which wipe off powder particles adhered to the inner walls of the device, thereby aiding dose emission. Sweeper lactose particles are larger than the cutoff value of the Cyclops, so generally, they stay in the device and are not inhaled [18]. ^2^ Determined in-house. N/A = not applicable.

**Table 2:** Pharmacokinetic parameters after the inhalation of Inbrija (84 mg) and Levodopa Cyclops (45, 90, and 135 mg) by fasted, healthy volunteers (*n* = 26). C_max_, T_max_, and t_1/2_ are reported as medians (Q1–Q3); AUC_0–4_, AUC_0–t_, and AUC_0–∞_ are reported as means with standard deviations.

	Inbrija 84 mg	Levodopa Cyclops 45 mg	Levodopa Cyclops 90 mg	Levodopa Cyclops 135 mg
C_max_ (ng/mL)	1017 (736–1433)	537 (426–657)	948 (823–1266)	1621 (1268–1834)
T_max_ (min)	20 (13–40)	24 (12–31)	23 (15–38)	15 (9–29)
AUC_0–4_ (ng⋅h/mL)	1679 (SD = 500)	876 (SD = 175)	1682 (SD = 364)	2618 (SD = 476)
AUC_0–t_ (ng⋅h/mL)	1955 (SD = 599)	1032 (SD = 187)	1976 (SD = 484)	3091 (SD = 522)
AUC_0–∞_ (ng⋅h/mL)	2006 (SD = 591)	1059 (SD = 191)	2029 (SD = 482)	3167 (SD = 538)
t_1/2_ (h)	1.35 (1.28–1.55)	1.49 (1.40–1.61)	1.47 (1.37–1.51)	1.43 (1.33–1.53)

## Data Availability

The original contributions presented in this study are included in the article/Appendix A. Further inquiries can be directed to the corresponding author.

## Abbreviations

The following abbreviations are used in this manuscript:

DPI	Dry powder for inhalation
NGI	Next generation impactor
OIDP	Orally inhaled drug product
C_max_	Maximum plasma concentration
T_max_	Time to maximum plasma concentration
AUC_0–4_	Area under plasma concentration-time curve from zero to four hours post-dose
AUC_0–t_	Area under the plasma concentration-time curve from zero to the last observed concentration
AUC_0–∞_	Area under plasma concentration-time curve from zero to infinity
t_1/2_	Plasma concentration half-life
SD	Standard deviation
